# Predictors of real-life mobility in community-dwelling older adults: an exploration based on a comprehensive framework for analyzing mobility

**DOI:** 10.1186/s11556-019-0225-2

**Published:** 2019-11-03

**Authors:** Eleftheria Giannouli, Michelle Pasquale Fillekes, Sabato Mellone, Robert Weibel, Otmar Bock, Wiebren Zijlstra

**Affiliations:** 1 Institute of Movement and Sport Gerontology, German Sport University Cologne, Am Sportpark Müngersdorf 6, 50933 Cologne, Germany; 2 University Research Priority Program ‘Dynamics of Healthy Aging’, University of Zurich, Andreasstrasse 15, 8050 Zurich, Switzerland; 3 Department of Geography, University of Zurich, Winterthurerstrasse 190, 8057 Zurich, Switzerland; 4 Department of Electrical, Electronic, and Information Engineering, University of Bologna, Viale Risorgimento 2, 40136 Bologna, Italy; 5 Institute of Physiology and Anatomy, German Sport University Cologne, Am Sportpark Müngersdorf 6, 50933 Cologne, Germany

**Keywords:** Smartphones, Life-space, physical activity

## Abstract

**Background:**

Reduced mobility is associated with a plethora of adverse outcomes. To support older adults in maintaining their independence, it first is important to have deeper knowledge of factors that impact on their mobility. Based on a framework that encompasses demographical, environmental, physical, cognitive, psychological and social domains, this study explores predictors of different aspects of real-life mobility in community-dwelling older adults.

**Methods:**

Data were obtained in two study waves with a total sample of *n* = 154. Real-life mobility (physical activity-based mobility and life-space mobility) was assessed over one week using smartphones. Active and gait time and number of steps were calculated from inertial sensor data, and life-space area, total distance, and action range were calculated from GPS data. Demographic measures included age, gender and education. Physical functioning was assessed based on measures of cardiovascular fitness, leg and handgrip strength, balance and gait function; cognitive functioning was assessed based on measures of attention and executive function. Psychological and social assessments included measures of self-efficacy, depression, rigidity, arousal, and loneliness, sociableness, perceived help availability, perceived ageism and social networks. Maximum temperature was used to assess weather conditions on monitoring days.

**Results:**

Multiple regression analyses indicated just physical and psychological measures accounted for significant but rather low proportions of variance (5–30%) in real-life mobility. Strength measures were retained in most of the regression models. Cognitive and social measures did not remain as significant predictors in any of the models.

**Conclusions:**

In older adults without mobility limitations, real-life mobility was associated primarily with measures of physical functioning. Psychological functioning also seemed to play a role for real-life mobility, though the associations were more pronounced for physical activity-based mobility than life-space mobility. Further factors should be assessed in order to achieve more conclusive results about predictors of real-life mobility in community-dwelling older adults.

## Background

Intact mobility is fundamental to healthy aging as it enables older adults to lead active and independent lives. Unfortunately, mobility consistently decreases with advancing age [1, 2], and such reductions have been linked to many adverse consequences for health and functioning. For example, reduced mobility has been linked to low quality of life [3], cognitive decline [4, 5], physical disability [6], falling [7], loss of independence, institutionalization [8], poor health status [9] and ultimately death [10–13].

Given the significance of mobility for independent functioning and the adverse effects of a reduced mobility on health, it is important to have knowledge of factors that have a negative impact on real-life mobility of older adults. Such knowledge may provide input for the further development of interventions aiming to prevent age-related mobility restrictions.

Real-life mobility comprises in-home mobility as part of domestic activities, as well as out-of-home mobility, such as purchasing daily necessities, visiting neighborhood facilities for healthcare or recreation as well as keeping up social relations. Thus, mobility in real-life may include the use of assistive devices (e.g. walking aids) as well as passive means of transportation, such as trains and cars. The above examples illustrate that the extent to which someone is mobile does not only depend on the physical ability to be mobile [14], but that mobility is a complex, multifaceted construct [15] with many potential influencing factors. Literature typically distinguishes two aspects of real-life mobility. One aspect relates to physical activity (i.e. any bodily movement produced by skeletal muscles that requires energy expenditure [16]. This physical activity-based mobility is typically assessed by actigraphy, step counters, or questionnaires. The other aspect is life-space mobility, which refers to the out-of-home/outdoor spatial mobility and includes physical activity-based mobility as well as passive means of transportation, such as trains and cars. Life-space mobility is determined by GPS or questionnaires.

In 2010, Webber et al. [17] introduced a framework including five categories of determinants (cognitive, psychosocial, physical, environmental and financial) which all potentially can influence mobility. Based on a variety of approaches, a number of studies have demonstrated associations of real-life mobility and aspects of physical functioning, such as muscle strength [18, 19], cardiovascular fitness [20], gait [21, 22], functional mobility and balance [14, 19, 23]; cognitive factors, such as global cognitive status [4, 24], or domain-specific cognitive functions such as working memory [25, 26], visuospatial attention [27, 28] and executive functioning [26, 29]; psychological factors such as control beliefs [30], outdoor motivation/goals [31, 32], spontaneity [33], depressive symptoms [34], fear of falling [35] and social factors such as social network size [36] and social involvement [37]. Moreover, real-life mobility is associated with socio-demographic factors such as age, gender, income, education [1, 2, 38], as well as environmental conditions such as size of living environment [39] and proximity to relevant destinations [40] or weather conditions [41–43].

These previous studies all investigated aspects of the relationships between real-life mobility and its influencing factors. However, none of the studies presented a comprehensive approach and a number of limitations can be identified: First of all, most of them relied on subjective mobility assessments (i.e. retrospective self-reports/questionnaires) [44] which are susceptible to recall bias due to memory issues and social desirability bias (overreporting good behavior) [45, 46] or, as mentioned above, have assessed factors from a single domain of functioning (e.g. only physical or only cognitive), failing to deliver clear-cut conclusions about the extent of contribution that each domain makes to real-life mobility. In addition to this, they have often focused on vulnerable populations with multiple risk factors for mobility restrictions, such as mild cognitive impairment [22, 47] and Parkinson’s disease [48] or have looked into single aspects of real-life mobility, e.g. only physical activity [31] or only life-space mobility [49]. However, mobility measures that reflect a person’s own activity and mobility measures which reflect life-space mobility may be sometimes related [50] but in essence they are different constructs which consequently may have differential associations with the various domains of functioning. Thus, studies should take this into account and assess all aspects of real-life mobility.

Aiming for a comprehensive approach based on the framework introduced by Webber et al. [17], this study examined associations of potential predictors from multiple domains with real-life mobility in older adults without severe physical and/or cognitive impairments. Our analysis includes different aspects of objectively assessed real-life mobility; measures that reflect a person’s physical activity-based mobility as well as measures that reflect life-space mobility. We hypothesize that physical activity-based mobility will correlate mainly with measures from the physical and psychological domain (rather than the cognitive domain [51]). Life-space mobility, in contrast, (especially measures such as life-space area and maximum action range) captures additional aspects of movement which may include the use of passive means of transportation such as cars, or buses/trams for urban transportation and trains/airplanes for transportation to regions beyond one’s city/town of residence, which do not necessarily require high levels of physical capacity, as well as motivational aspects. Since vehicle-related skills such as driving, and use of public transportation require high levels of visuospatial processing (to avoid collisions) [52], executive functioning (to plan routes), and memory (to remember destinations) [25], we hypothesize that life-space mobility will correlate mainly with measures from the cognitive and psychosocial domain (e.g. self-efficacy and social network size) rather than the physical domain.

To the best of our knowledge, this is the first study to simultaneously take into account physical, cognitive, psychological, social, environmental and demographical measures in analyzing predictors of real-life mobility, as measured objectively based on physical activity-based mobility as well as life-space mobility, in older adults without severe mobility limitations and/or cognitive impairments.

## Methods

This study was run in two waves; Wave 1: February–September 2014 and Wave 2: May–December 2016. All participants underwent an ambulatory mobility assessment over one week (on average) as well as a laboratory-based test battery divided into two sessions. The ambulatory mobility assessment was the same for all participants. The multi-domain, laboratory-based assessment included several physical, cognitive, social and psychometric tests which partly differed between Wave 1 and Wave 2. The local ethics committee approved the study protocol (Wave 1 Registration Nr: 5/2014 and Wave 2 Registration number: 38/2015) which is in accordance with the declaration of Helsinki.

### Participants

Community-dwelling older adults were recruited primarily by handing-out information brochures and holding presentations about the study at local senior citizen gatherings. In total, 157 persons (87 for Wave 1 and 70 for Wave 2) meeting the criteria for participation in the study were initially recruited. Exclusion criteria were: age younger than 60 years, suffering from any serious diseases which could interfere with functional mobility and inability to stand up from a chair independently. All participants signed an informed consent agreeing to participate in the study. Because the test battery of Wave 1 included a stress-test, participants of Wave 1 had to provide a physician’s written statement of non-objection for this person to participate.

### Real-life Mobility assessment

Mobility in real life was assessed over one week based on motion (IMU) and positioning (GPS) data from smartphones. The detailed procedure for the ambulatory mobility assessment has been described elsewhere [14]. In short, participants were given a smartphone (Samsung Galaxy SIII™) in an elastic belt which they wore around their waist all day and took off only while sleeping and showering. The real-life data recording took place between the first laboratory session, in which participants received the smartphone, and the second session, in which they returned it. We aimed to record participants’ real-life mobility for 7 days.

From the IMU data following two variables were calculated [53]: ‘Active- & Gait Time’ (AGT) [h], defined as the sum of active and gait intervals with an intensity higher than 3 METs; and ‘Number of steps’ (Steps). Since data collection did not target full 24-h periods and registration times differed between days and participants, we adjusted the data by excluding registration periods before 7.00 AM and after 9.00 PM as well as registration periods shorter than 9 h; In order to have consistent assessments for all participants, AGT and Steps scores were then scaled to fit a 12-h day and were subsequently averaged across all registration days of a given participant. Scaled values were calculated as follows: (Actual AGT value for each day * 12) divided by (total registration hours for all activity types).

**Fig. 1:**
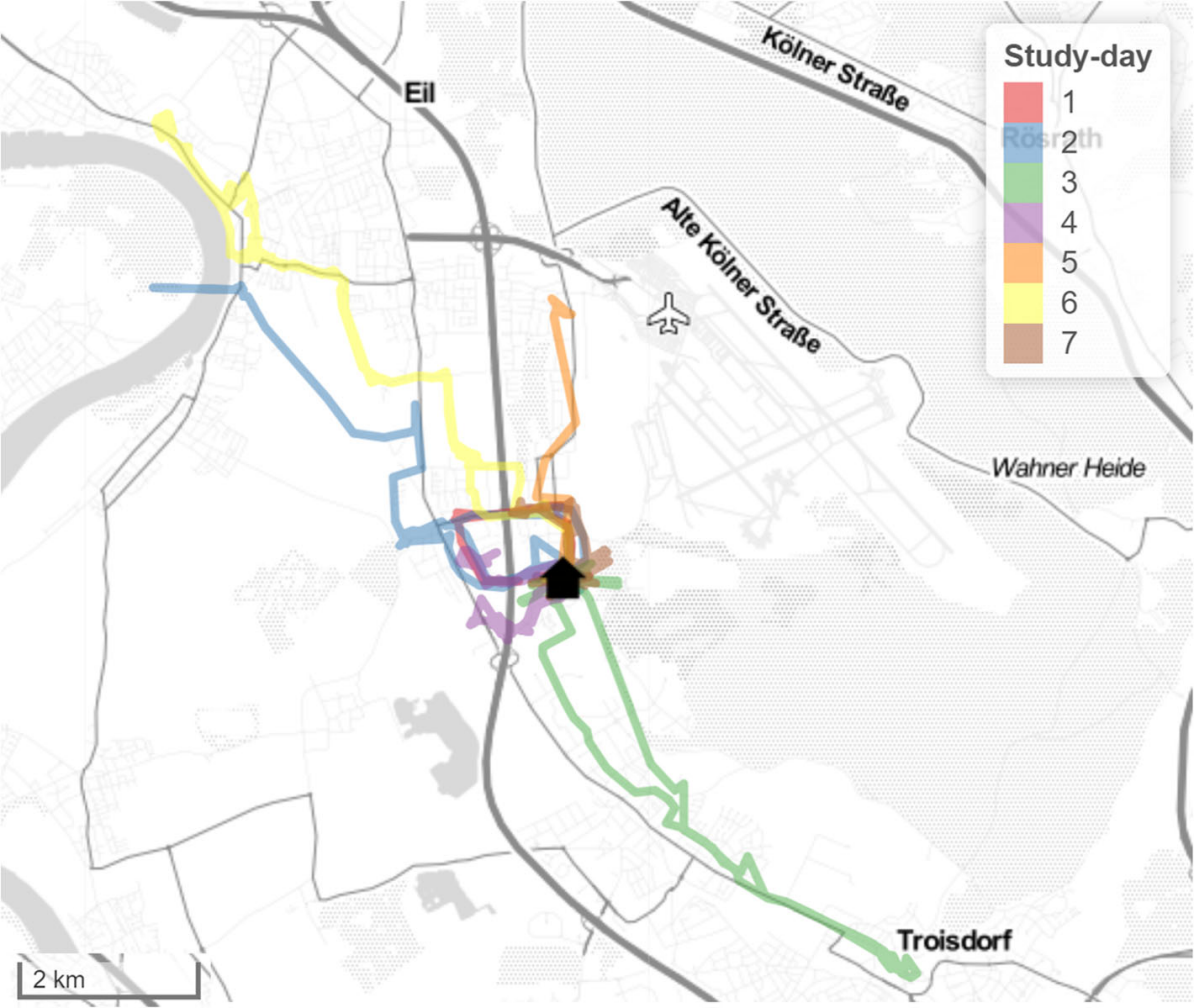
Sample GPS trajectories of one participant. *Note: Each color represents trajectories for different days and the black house symbol represents home location)*

From the GPS data following three variables were calculated: based on the R ‘chull’ function, ‘Life-space area’ [km^2^] was calculated as the convex hull of all GPS coordinates that were obtained during one day. The available daily life-space area values were then averaged to obtain (average) life-space area per day for each participant; ‘total Distance’ [km], defined as the average daily displacement of a person during the registration period; and ‘Maximum action range’ (AR-max) [km], defined as the largest straight-line distance away from the home location during the registration period. In order to focus on habitual mobility and exclude exceptional trips/extreme values, only data within 15 km around the participants’ home (comparable to the size of the greater area of Cologne, Germany, where the study took place) were included in the analysis [14, 54]. Since GPS reception is usually not possible indoors, the life-space mobility measures primarily cover outdoor mobility. Contrary to the physical activity-based measures, they also cover passive modes of transportation. Figure 1 presents a typical example of GPS data obtained over 7 recording days.

### Multi-domain measures

Three standard demographic measures (age, gender and education) as well as maximum temperature (averaged across all days for each participant’s registration period) were used as predictor variables for both study waves. Maximum temperature was included as a weather (environmental) measure since it is known to affect mobility levels [41, 55].

In addition to this, three relevant predictors were chosen for each of the four (physical, cognitive, psychological and social) domains of functioning. Each predictor was operationalized by one to four domain-specific measures. Table 1 presents all multi-domain assessments that were administered in Wave 1 and Wave 2.

**Table 1:** Laboratory-based assessments for wave 1 and wave 2

	Wave 1		Wave 2	
Domains	Predictors	Measures (Assessment tools)	Predictors	Measures (Assessment tools)
Physical	Gait	Gait Speed, iTUG [56]	Gait	Gait Speed
	Muscle strength	Handgrip Strength, Leg Strength	Muscle strength	Handgrip Strength, Leg Strength
	Cardio fitness	PWC 130	Balance	Jerk, 4SST [57]
Cognitive	Planning ability	HOTAP.A Test [58]	Planning ability	HOTAP.A Test [58]
	Visuospatial attention	Attention-Window (AW) test [59]	Visuospatial attention	Attention-Window (AW) Test [59]
	Spatial working memory	Grid-Span [60]	Switching	TMT, D2 Test of Attention [61]
Psychological	Self-efficacy	*FES* [62]*,* GSE [63]	Self-efficacy	FES [64], ABC-D [62], SSE [65], mGES-D [66]
	Rigidity	MPTE.2 [67]	Loneliness	UCLA Loneliness Scale (UCLA) [68]
	ArousalX	MPTE.1 [67]	Depression	GDS [69]
Social	Sociableness	MPTE.3 [67]	Sociableness	MPTE.3 [67]
	Perceived Help Availability	ISEL-TSS [70]	Perceived Help Availability	ISEL-TSS [70]
	Perceived Ageism	ASS [71]	Social Networks	LSNS [72]

*Note: iTUG = instrumented Timed Up-and-Go Test, PWC=Physical Work Capacity Test, HOTAP = Action- and Daily planning, FES=Falls Efficacy Scale, GSE = General Self-Efficacy Scale, MPTE = Multidimensional Personality Test for Adults, ISEL-TSS = Interpersonal Support Evaluation List-Tangible Support Subscale, ASS = Ageism Survey Scale, 4SST = Four Square Step Test, TMT = Trail-Making-Test, ABC-D = German version of the Activities-Specific Balance Confidence, SSE = Stair Self-Efficacy Scale, mGES = modified Gait Efficacy Scale, GDS = Geriatric Depression Scale, LSNS = Lubben Social Network Scale*

### Statistical analyses

The variables ‘Life-space area’, ‘total Distance’ and ‘AR-max’ were square-root-transformed to achieve normal distribution. Outlier cases (0.88%) were identified using Tukey’s outlier filter [73] and removed. Missing data (0–4.3% for the Wave 1 variables and 0–4.7% for the Wave 2 variables) were imputed using the pertinent mean values. To make sure that the imputed dataset was not biased, we applied Little’s MCAR test, which showed that data were missing randomly. In order to have a first look into the overall relationships between real-life mobility and the various multi-domain measures we performed a multiple correlation analysis (Spearman’s rank correlation coefficients). Afterwards, a series of stepwise multiple regression analyses were applied, in which the multi-domain factors that were significantly correlated with real-life mobility served as predictors and the real-life mobility measures served as dependent variables. For the stepwise models the limit was F = 0.10 for removal and F = 0.05 for entry of variables. Normality of the residuals was checked using the Shapiro-Wilk test. For all analyses, the significance level was set at 0.05; the only exception was the Kolmogorov–Smirnov test for the normality of datasets, for which the significance level was set at 0.10.

Correlation as well as the regression analyses were first performed for the two study waves separately and then the common measures were used to complete these analyses with a pooled dataset (*n* = 154) from both studies.

## Results

### Descriptive statistics

From the 157 participants, three were excluded from the analysis: one for dropping out of the study after the first test session and two for not completing the ambulatory mobility assessment. The final sample, therefore, consisted of 85 persons for Wave 1 and 69 persons for Wave 2. As mentioned earlier in the methods section, we aimed for a 7-days mobility assessment. Unfortunately, it was not always possible to organize the sessions exactly 7 days apart. However, only few participants (*n* = 9 for wave 1, and *n* = 17 for wave 2) had a different number of days (6, 8, 9, or) of mobility registration. In all other participants exactly 7 days were available. Hence, rather than substantially reducing the available complete real-life datasets, and facing the challenge of defining rational criteria for selecting 6 days (out of the 7, 8 or 9), we decided to use total registration times that ranged from 6 to 9 days.

Table 2 provides a description of the samples’ demographics as well as real-life mobility measures. Of all reported real-life measures, only ‘Total Distance’ values were significantly different between wave 1 and wave 2, therefore, they were excluded from further analysis.

**Table 2:** Demographic and real-life mobility descriptive data for the two study waves, mean ± SD or %

	Wave 1	Wave2
Age (years)	72.3 ± 5.9	69.5 ± 4.9
Women	62%	62%
age	72.4 ± 5.8	69.0 ± 4.9
Men	38%	38%
age	72.4 ± 5.9	70.3 ± 5.0
BMI (kg/m^2^)	24.3 ± 3.45	25.3 ± 4.07
IPAQ (score)	7403 ± 5840	7370 ± 6539
Living in assisted living facilities	15%	0%
Gait assistance	0%	0%
Living alone	50%	39%
Higher education degree	18%	26%
Fallers	19%	40%
Reported health problems	77%	93%
Multimorbid	43%	69%
Exercising regularly	78%	77%
*Real-life measures*		
Mean registration days	7.6 ± 1.9	6.7 ± 1.6
AGT [h]	1.07 ± 0.51	1.13 ± 0.51
Steps (n)	9604 ± 3539	10,323 ± 3485
Life-Space area [km^2^]	11.7 ± 17.1	13.6 ± 0.1
Total Distance [km]	23.6 ± 17.3	40.3 ± 26.2
AR-max [km]	9.44 ± 4.9	9.70 ± 4.2

Table 3 summarizes the descriptive data of the multi-domain assessments for the two study waves.

**Table 3:** Descriptive data for Wave 1 and Wave 2

	Wave 1		Wave 2	
Domain	Measures	Mean (±SD)	Mean (±SD)	Measures
Physical	Grip Strength [N]	281.65 (±111.72)	283.84 (±114.85)	Grip Strength [N]
	Leg Strength [*Kg*_*NE*_*/Kg*_*BW*_]	1.43 (±0.45)	1.48 (±0.46)	Leg Strength [*Kg*_*NE*_*/Kg*_*BW*_]
	PWC	1.61 (±0.88)	0.0858 (±0.059)	Jerk [m/s^*3*^]
	iTUG [s]	16.01 (±2.61)	7.73 (±1.66)	4SST
	Gait speed [m/s]	1.35 (±0.16)	1.42 (±0.14)	Gait speed [m/s]
Cognitive	HOTAP.A	10.3 (±4.59)	13.3 (±4.33)	HOTAP.A
	Attention Window	69.3 (±22.54)	32.1 (±11.16)	Attention Window
	Grid-Span	3.85 (±0.93)	144.5 (±44.01)	d2
			50.14 (±24.57)	TMT (B-A)
Psychological	FES	19.34 (±4.04)	19.35 (±4.39)	FES
	GSE	33.05 (±4.93)	83.48 (±22.32)	SSE
	MPTE.1	8.02 (±2.06)	90.56 (±12.91)	ABC
	MPTE.2	9.41 (±2.34)	89.2 (±15.74)	mGES-D
			54.07 (±4.26)	UCLA
			1.39 (±1.68)	GDS
Social	MPTE.3	2.56 (±2.2)	2.44 (±1.91)	MPTE.3
	ISEL-TSS	20.24 (±3.81)	21.13 (±3.4)	ISEL-TSS
	ASS	4.79 (±6.73)	33.1 (±7.4)	LSNS

*Note: Kg*_*NE*_*/Kg*_*BW*_ *= Knee extensor strength (kg) normalized for body weight (kg), B-A = points for part B of the Trail-Making Test minus points for part A of the Trail-Making Test, iTUG = instrumented Timed Up-and-Go Test, PWC=Physical Work Capacity Test, HOTAP = Action- and Daily planning, FES=Falls Efficacy Scale, GSE = General Self-Efficacy Scale, MPTE = Multidimensional Personality Test for Adults, ISEL-TSS = Interpersonal Support Evaluation List-Tangible Support Subscale, ASS = Ageism Survey Scale, 4SST = Four Square Step Test, TMT = Trail-Making-Test, ABC-D = German version of the Activities-Specific Balance Confidence, SSE = Stair Self-Efficacy Scale, mGES = modified Gait Efficacy Scale, GDS = Geriatric Depression Scale, LSNS = Lubben Social Network Scale*

### Correlation analyses

#### Wave 1

Table 4 illustrates that all multi-domain assessments except education, maximum temperature, general self-efficacy, MPTE-3 and ISEL-TSS had significant associations with at least one of the real-life measures. Leg strength correlated significantly with all four of the real-life measures but iTUG showed in average the strongest correlations. General self-efficacy (GSS) correlated significantly with only one real-life measure, and Grid-Span showed the weakest correlations. Overall, correlation coefficients were low to moderate [74].

**Table 4:** Correlation coefficients r_s_ between all real-life mobility measures and multi-domain measures for Wave 1. (∗*p* < .05; ∗∗*p* < .01)

Domain		AGT	Steps	Life-Space area	AR-max
	Age	−.332**	−.124	−.256**	−.256**
	Gender	.148	.146	.189*	.097
	Education	−.059	−.093	.113	.038
	Maximum Temperature	−.097	−.133	−.169	−.129
Physical	Grip Strength	.251*	.121	.329**	.184*
	Leg Strength	.336**	.274**	.286**	.263**
	Physical Work Capacity Test	.17	.258**	−.079	.021
	iTUG	−.429**	−.237*	−.161	−.087
	Gait Speed	.346**	.201*	.146	.067
Cognitive	HOTAP.A	.266**	.028	.250*	.257**
	Attention Window	.332**	.137	.249*	.209*
	Grid-Span	.194*	.128	.204*	.180*
Psychological	Falls Efficacy Scale	−.229*	−.126	−.093	−.191*
	General Self-Efficacy	−.101	−.137	−.094	−.096
	MPTE.1 (arousal)	−.301**	−.267**	−.029	−.162
	MPTE.2 (rigidity)	−.390**	−.382**	−.055	−.109
Social	MPTE.3 (sociableness)	−.035	.002	.127	.073
	ISEL-TSS	.132	.032	.145	.146
	Ageism Survey Scale	−.246*	−.261**	−.119	−.186*

*Note: iTUG = instrumented Timed Up-and-Go test, HOTAP = Action- and Daily planning,, MPTE = Multidimensional Personality Test for Adults, ISEL-TSS = Interpersonal Support Evaluation List-Tangible support subscale*

#### Wave 2

Similar to Wave 1, there were six measures from Wave 2 (maximum temperature, HOTAP.A, Attention Window as well as all the social measures) that did not show significant correlations with real-life measures. The measure which showed the most significant correlations with real-life measures was grip strength, correlating significantly with all four of the real-life measures. However, the strongest correlations were found for the 4SST. The correlation coefficients were low to moderate for this wave as well. Table 5 illustrates all bivariate correlation coefficients between the predictor measures and the real-life measures.

**Table 5:** Correlation coefficients r_s_ between all real-life mobility measures and multi-domain measures for Wave 2. (∗*p* < .05; ∗∗*p* < .01)

Domain		AGT	Steps	Life-Space area	AR-max
	Age	−.397**	−.177	−.043	−.101
	Gender	−.09	−.036	.340**	.349**
	Education	.372**	.285**	.031	.12
	Maximum Temperature	−.007	−.019	−.099	−.077
Physical	Grip Strength	.319**	.283**	.201*	.205*
	Leg Strength	.065	−.007	.240*	.239*
	Jerk	−.314**	−.184	−.072	−.035
	Four Square Step Test	−.431**	−.218*	−.038	.011
	Gait Speed	.218*	.197	.028	.056
Cognitive	HOTAP.A	.198	.037	.05	.068
	Trail Making Test (B-A)	−.306**	−.177	−.02	−.05
	d2 Test of Attention	.201*	.082	.155	.148
	Attention Window	−.006	−.07	.002	.084
Psychological	Falls Efficacy Scale	−.206*	−.19	.072	.410*
	Stair Self-Efficacy Scale	.277*	.217*	−.001	.006
	ABC-D	.265*	.212*	.001	−.005
	mGES-D	.229*	.211*	−.018	.077
	UCLA loneliness scale	−.074	−.117	−.037	−.025
	Geriatric Depression Scale	−.234*	−.267*	.101	.02
Social	Lubben Social Network Scale	.005	−.111	.047	.149
	MPTE3	−.132	−.176	.061	−.027
	ISEL-TSS	.001	.054	.073	.136

*Note: ABC-D = German version of the Activities-Specific Balance Confidence, mGES = modified Gait Efficacy Scale, MPTE = Multidimensional Personality Test for Adults, LSNS = Lubben Social Network Scale*

#### Pooled dataset

Table 6 summarizes the results of the correlation analysis for the pooled dataset. Grip strength and leg-strength show the largest number and strongest correlations with real-life mobility measures, which hints at the importance of physical measures for real-life mobility overall.

**Table 6:** Correlation coefficients r_s_ between all real-life mobility measures and multi-domain measures for the pooled dataset. (∗*p* < .05; ∗∗*p* < .01)

Domain		AGT	Steps	Life-Space area	AR-max
	Age	−.366**	−.166*	−.191**	−.194**
	Gender	.033	.064	.233**	.194**
	Education	.173*	.13	.072	.003
	Max. Temperature	−.05	−.08	−.102	−.102
Physical	Grip Strength	.276**	.181*	.251**	.191**
	Leg Strength	.265**	.216**	.221**	.250**
	Gait speed	.233**	.138*	.108	.117
Cognitive	HOTAP.A	.221**	.051	.216**	.176*
	Attention Window	.023	−.052	−.021	.088
Psychological	Falls Efficacy Scale	−.217**	−.147*	−.01	−.088
Social	MPTE3	−.076	−.077	.048	.035
	ISEL-TSS	.087	.052	.132	.142*

*Note: MPTE = Multidimensional Personality Test for Adults, ISEL-TSS = Interpersonal Support Evaluation List-Tangible support subscale*

#### Regression analyses

To evaluate the predictive ability of the domain specific measures for each of the real-life measures four stepwise multiple regression analyses were conducted for each dataset (Wave 1, Wave 2 and pooled dataset). The predictor variables were checked for multicollinearity, and all variance inflation factor values were below 1.117 indicating no multicollinearity among factors. Their results are summarized in Table 7.

**Table 7:** Predictors with their standardized regression coefficients and the adjusted coefficients of determination for the physical activity-based and the life-space models of Wave 1, Wave 2 and the pooled dataset (∗*p* < .05; ∗∗*p* < .01)

Physical activity-based mobility				Life-space mobility			
AGT		Steps		Life-Space area		AR-max	
Predictors	Beta	Predictors	Beta	Predictors	Beta	Predictors	Beta
*Wave 1*							
iTUG.	−.362*	MPTE.2	−.371**	Leg Strength	.271*	Leg Strength	.266*
MPTE.2	−.325**	Leg Strength	.221*			Age	−.341*
R^2^ = .280**		R^2^ = .183**		R^2^ = .063*		R^2^ = .142*	
*Wave 2*							
Age	−.294*	mGES-D	.310*	Grip Strength	.341*	FES	−.299*
Education	.325**	Education	.235*				
SSE	.277*						
R^2^ = .303**		R^=2^ = .136*		R^2^ = .103*		R^2^ = .076*	
*Pooled dataset*							
Age	−.287**	Leg Strength	.232*	Grip Strength	.297**	Grip Strength	.244*
Leg Strength	.201*					Age	−.198*
R^2^ = .142**		R^2^ = .048*		R^2^ = .082**		R^2^ = .113**	

The best predictive model (i.e. the model with the highest adjusted coefficient of determination) for all three datasets was AGT (R^2^ between 14 and 30%), which had three significant predictors for Wave 2 and two significant predictors for Wave 1 and the pooled dataset, respectively. Most of the regression models had one or two significant predictors. ‘AGT’ of Wave 2 had three predictors. The predictor measures explained a somewhat larger proportion of variance in real-life measures for Wave 1 (between 6 and 28%) and Wave 2 (between 8 and 30%) compared to the pooled dataset (between 5 and 14%).

Strength measures (and especially leg strength) were retained in all but four of the regression models across all datasets. Adding iTUG, which was retained in another one model, it is clear that measures from the physical domain were the most important for real-life measures. Demographic variables also seem to be important for real-life mobility, especially age, which was retained in four regression models followed by education, which was retained in two physical activity-based models (AGT and Steps). Neither cognitive nor social measures were retained in any of the models. Psychological measures (rigidity and self-efficacy measures) were retained in five regression models from Wave 1 and Wave 2. Overall, the analyses indicated that the predictors accounted for a significant but low proportion of variance (between 5 and 30%) in real-life measures.

## Discussion

In the current work we aimed to examine the predictive value of demographic and environmental factors, as well as factors from four domains of functioning (physical, cognitive, psychological, social), for real-life mobility of community-dwelling older adults. Our data provide support for our first hypothesis that physical activity-based mobility is associated with physical and psychological functioning, but do not support our second hypothesis that life-space mobility is more associated with socio-cognitive functioning.

Physical measures, and especially strength, showed the highest associations with real-life mobility (both for physical activity-based mobility as well life-space mobility). This is in line with previous epidemiological studies that have reported associations between physical activity [18], or life-space mobility [33], with several aspects of physical functioning. Since our GPS analysis didn’t differentiate between active and passive modes of transportation, it may be that our participants made little use of passive transportation and instead were mainly physically active, which would explain the clear associations of physical and not so much of cognitive and psychosocial measures with life-space mobility.

Contrary to our expectations, cognitive and social functioning showed no associations with real-life mobility. Although a plethora of cross-sectional [27, 36, 75] as well as longitudinal studies [76] show significant associations, discrepant results have also been reported [77, 78]. Regarding cognition, models with cognitive measures as a sole predictor are usually significant; however, adding other predictors often renders cognition non-significant [34]. It is possible that the role of cognition for real-life mobility, and especially life-space mobility, would be different/more important, if participants with cognitive impairments were included in the study. Also, some of the strongest and most usual intervening variables modifying the relationship between mobility and cognition are psychological measures; studies have reported on the interplay and mediating role of several psychological measures such as depression [34] or motivational resources [29] for the association between mobility and cognition, however again with mixed results. The importance of psychological measures especially that of rigidity (MPTE.2) and self-efficacy for mobility in old age is also evident in our results, though much more pronounced for physical activity-based mobility than life-space mobility. MPTE.2 remained as a significant predictor in the models of AGT and steps. This is in line with Tung et al. [22] as well as Tanaka & Yamagami [33], who reported significant association between life-space and apathy as well as spontaneity, both constructs very similar to rigidity. Furthermore, the importance of self-efficacy for real-life mobility is depicted in the results of Wave 2, which are in accordance with Amireault et al. [31] and Bauman et al. [79], as well as Auais et al. [35], highlighting the role of self-efficacy for physical activity as well as life-space. Interestingly, compared with Wave 1, self-efficacy measures were much more associated with mobility in Wave 2. This can be explained by the fact that for Wave 2 self-efficacy was assessed much more comprehensively, both in terms of number of measures (two measures used in wave 1 and four measures used in wave 2) as well as in terms of their specificity (wave 1 included a general self-efficacy scale, whereas wave 2 included only mobility-related self-efficacy measures). According to Bandura [80] there is no all-purpose measure of perceived self-efficacy; assessments should be tailored to the specific domain of functioning that is the object of interest. Indeed, mGES-D, a tool assessing self-efficacy specifically for walking, remained as a significant predictor for the ‘Steps’ model, whereas SSE, a tool assessing self-efficacy for stair climbing remained as a predictor for AGT, which covers a wider spectrum of activities than just walking, including stair-climbing.

While previous studies report strong associations between environmental (e.g. weather) factors [41] with real-life mobility, our results are in line with Ullrich et al. (2019) [47] who did not find any associations between life-space mobility and several weather parameters. In our dataset maximum temperature did not show any correlations with any of the real-life measures although this specific weather measure has shown to be one of the best predictors of physical activity in older people [55] and although there was enough variation in maximum temperature on the days of data collection periods (Min = 10.6 °C, Max = 29.5 °C, Mean = 21.5 °C, SD = 3.7 °C). Therefore, the role of additional weather measures for real-life mobility, such as precipitation and sunshine hours, should be assessed further.

Finally, two of the three of the assessed demographic measures showed associations with real-life measures; gender did not seem to affect real-life mobility. Age was associated with both physical activity-based as well as life-space measures, whereas education only with physical activity measures. The latter can be explained by the fact that highly educated individuals are more likely to remain physically active due to their awareness of the positive effects of an active lifestyle for health and quality of life.

Although the current study has the strength of examining the relationship between objectively measured physical activity-based and life-space measures with a wide range of potential multi-domain mobility-influencing factors, there are still other important factors that potentially have a large influence on real-life mobility, such as (perceived) walkability of the neighborhood, proximity and accessibility of points of interest, neighborhood satisfaction [81] and personality traits [37], which were not assessed in this study. This possibly explains the rather low proportion of variance that our predictors explained for real-life mobility overall, and especially for life-space mobility measures. However, despite a lower proportion of explained variance, our results are similar to the only study that has used Webber’s comprehensive framework [17] to look into multidomain determinants for life-space mobility in older adults with cognitive impairments [47] which found that physical and psychosocial measures (and not cognitive, financial, environmental, cultural and biographical measures) accounted for 36% in life-space mobility. Further limitations include the cross-sectional design of this study, which does not allow causal conclusions, the sample size and type, which was limited to rather high-functioning and active older adults, prohibiting the generalization of this study’s results to other populations as well as the two different samples with somewhat large differences in some of the common functioning measures (e.g. Attention Window) as well as background measures which might have affected the results of the pooled data analyses. Finally, the correlation values have not been corrected for multiple testing, thus, there might be false positives in the variable selection for the regression analyses.

Future studies should examine other types of samples, e.g. frail or inactive individuals and apply longitudinal designs looking into the change of real-life mobility and the remainder of potential influencing factors over time, preferably also factors that have not been assessed in this study such as (perceived) walkability of the neighborhood, proximity and accessibility of points of interest, and overall neighborhood satisfaction, as well as several skills like driving, access to a vehicle etc.

## Conclusions

Given its limitations and its exploratory character, this study cannot provide a definitive answer regarding the factors that predict physical activity and life-space mobility. However, it provides first important insights on the relative contribution of multi-domain factors for real-life mobility. Primarily physical, but also psychological factors, were stronger predictors of real-life mobility (than cognitive and social). Therefore, they may be targets for interventions aiming to improve real-life mobility in community-dwelling older adults without severe mobility limitations. Specifically, such interventions could combine physical exercise with strengthening of self-efficacy, e.g., through social networks, motivation strategies or approaches based on Social Cognitive Theory [82]. However, there is still a large proportion of variance that remains unexplained, especially for life-space mobility, which indicates that there are other factors that play a role for real-life mobility in older adults which have not been assessed in this study which might also have implications for interventions that do not address functioning but rather unmodifiable/hard to modify factors such as urban planning.

## Data Availability

The datasets used and/or analyzed during the current study are available from the corresponding author on reasonable request.
